# Validating the use of intrinsic markers in body feathers to identify inter-individual differences in non-breeding areas of northern fulmars

**DOI:** 10.1007/s00227-016-2822-1

**Published:** 2016-02-29

**Authors:** Lucy R. Quinn, Andrew A. Meharg, Jan A. van Franeker, Isla M. Graham, Paul M. Thompson

**Affiliations:** Institute of Biological and Environmental Sciences, University of Aberdeen, Lighthouse Field Station, Cromarty, IV11 8YJ UK; Institute for Global Food Security, Queen’s University Belfast, 18-30 Malone Road, Belfast, BT9 5BN Northern Ireland, UK; IMARES Wageningen UR, PO Box 57, 1780 AB Den Helder, The Netherlands; British Antarctic Survey, High Cross, Madingley Road, Cambridge, CB3 0ET UK

## Abstract

Many wildlife studies use chemical analyses to explore spatio-temporal variation in diet, migratory patterns and contaminant exposure. Intrinsic markers are particularly valuable for studying non-breeding marine predators, when direct methods of investigation are rarely feasible. However, any inferences regarding foraging ecology are dependent upon the time scale over which tissues such as feathers are formed. In this study, we validate the use of body feathers for studying non-breeding foraging patterns in a pelagic seabird, the northern fulmar. Analysis of carcasses of successfully breeding adult fulmars indicated that body feathers moulted between September and March, whereas analyses of carcasses and activity patterns suggested that wing feather and tail feather moult occurred during more restricted periods (September to October and September to January, respectively). By randomly sampling relevant body feathers, average values for individual birds were shown to be consistent. We also integrated chemical analyses of body feather with geolocation tracking data to demonstrate that analyses of δ^13^C and δ^15^N values successfully assigned 88 % of birds to one of two broad wintering regions used by breeding adult fulmars from a Scottish study colony. These data provide strong support for the use of body feathers as a tool for exploring non-breeding foraging patterns and diet in wide-ranging, pelagic seabirds.

**Electronic supplementary material:**

The online version of this article (doi:10.1007/s00227-016-2822-1) contains supplementary material, which is available to authorized users.

## Introduction

Marine organisms are exposed to a variety of naturally occurring and anthropogenic chemicals. These have the potential to provide biogeochemical markers that can underpin studies of both spatial and foraging ecology (Ramos and González-Solís 2012). Wide-ranging marine top predators are likely to experience large spatial variation in the distribution of chemical tracers (Burger and Gochfield 2000; Gómez-Díaz and González-Solís 2007; Hobson and Bond 2012). Previous studies have used these markers to investigate contaminant levels (Bond and Lavers 2011; Moreno et al. 2011), diet (Hooker et al. 2001; Fisk et al. 2002) and migratory patterns (Hobson, 1999; Seminoff et al. 2012). However, detailed interpretation of these results ideally requires information on both where and when those chemicals were incorporated into the target tissues (Burger and Gochfeld 2000; Polizzi et al. 2013).

Analysis of intrinsic markers is particularly suited for seabirds, which can be sampled when they return annually to their breeding colonies (Furness and Camphuysen 1997). The different tissue types that may be sampled (e.g., feather, blood, muscle) represent different timescales over which the chemicals have been assimilated (Pearson et al. 2003; Bearhop et al. 2004). However, the distribution of birds breeding at particular colonies during the preceding winter is often uncertain. This is of particular importance when making colony-level inferences about foraging, because birds breeding at the same colony may exhibit individual differences in wintering areas (Kubetzki et al. 2009; Harris et al. 2010; Kopp et al. 2011). Thus, whilst stable isotopes have been commonly used to study winter foraging ecology (Cherel et al. 2006; Dehnhard et al. 2011), few studies have been based upon individuals with known foraging patterns (*cf.* Furness et al. 2006; Phillips et al. 2009; Jaeger et al. 2010; Leat et al. 2013). Recent advances in tracking technology now provide opportunities to validate these chemical-based methodologies for a wider range of species.

Feathers are generally the preferred tissue for studies of this kind because they are metabolically inert (Inger and Bearhop 2008; Hobson and Bond 2012) and relatively easily collected from breeding individuals (Bearhop et al. 2002). However, this requires information on the study species’ moult pattern to relate chemical results to a particular time period or location (Inger and Bearhop 2008). Whilst the pattern of moult in different feathers is well understood in many terrestrial species (Hinsley et al. 2003; Ryder and Wolfe 2009; Gargallo 2013), comparable data are rare from seabirds because they spend so much of the year at sea. Nevertheless, whilst moult in temperate and polar seabirds may commence towards the end of a reproductive attempt, most feather formation occurs within the non-breeding period (Quillfeldt et al. 2005; Bridge 2006; Allard et al. 2008). Most data for seabirds relate to the timing of moult in primary and secondary wing feathers (Ginn and Melville 1983) or for species with a unique, simultaneous body moult pattern (Carravieri et al. 2014). However, it is not possible to take whole wing feathers from live birds, and sub-samples of feather tips (Cherel et al. 2000) relate to relatively short periods of growth. Alternatively, chemical analyses of body feathers may provide a more general indication of foraging activity through the non-breeding period (Bearhop et al. 2006). However, for many species, limited understanding of the timing of moult and variability in the chemical composition of different body feathers constrains the extent to which chemical signatures can be used to explore winter foraging patterns (Larson and Hobson 2009).

Recent tracking data identified marked individual differences in wintering location within a North Atlantic colony of northern fulmars (hereafter fulmar, *Fulmarus glacialis*) (Quinn 2014). Furthermore, individual birds were distributed widely but tended to be consistent in their use of two broad-scale over-wintering areas (deep Atlantic waters, and relatively shallow waters over the continental shelf) that are likely to differ in their biogeochemistry. This study system provides a rare opportunity to integrate analyses of feather chemistry with seabird tracking data on winter distribution, to test for spatial differences in intrinsic markers. However, additional work is first required to assess whether chemical composition of body feathers is representative of the winter non-breeding period. Existing information on moult patterns in *Procellariformes* such as fulmars is limited, and based either on observations of birds at breeding colonies (Carrick and Dunnet 1954; Allard et al. 2008) or analysis of beached or by-caught individuals during the non-breeding season (van Franeker 2004). These limited data suggest that, in fulmars, adult moult begins post-breeding (late August) (Carrick and Dunnet 1954; Allard et al. 2008) and is be completed by the end of February (Ginn and Melville 1983).

This study aimed to validate the use of body feathers, specifically those which are easily collected from the ventral region, to assess individual and spatial variation in trace metals and stable isotope markers over the non-breeding period. If there is co-variation in chemical composition in wing feathers and body feathers, it would suggest growth of these different feathers occurs over a similar period. In contrast, if body feathers grow later in the winter, they may be more similar in composition to tail feathers, as fulmars are known to initiate tail moult when wing moult is around 75 % complete (Ginn and Melville 1983).

Using a combination of morphological, tracking and chemical analyses, the specific objectives were to: (a) assess seasonal variation in activity patterns to identify most likely period of moult, (b) compare moult timings and chemical loadings in wing, tail and body feathers, (c) assess within-individual variability in chemical loadings in body feathers, and (d) to validate the use of body feathers as a proxy for identifying broad scale wintering areas.

## Materials and methods

### Study site and logger deployment

Data on winter distribution, activity and feather chemistry were collected from adult fulmars breeding on Eynhallow, Orkney (59°8′N; 3°8′W). Fieldwork was conducted between 2006 and 2012 in parallel with ongoing population studies (see Lewis et al. 2009; Cordes et al. 2015), when approximately 100 active nests were recorded annually. Breeding adults were caught at the nest using a hand net or noose under licence from the British Trust for Ornithology, and British Antarctic Survey (BAS) light-based Global Location Sensor (GLS) loggers (Phillips et al. 2004) were attached to the darvic leg-rings of 163 birds using cable ties. The total device weight (including leg ring) was 3.6 g, representing <0.5 % of the lightest recorded fulmar’s body weight. Annual attendance of breeding adults in this study population varies with environmental conditions and may be as low as 50 % in some years (Thompson and Ollason 2001). This can constrain and delay logger recovery rates, which were 46 % over one year and 76 % over two or more years. After re-capture, a randomly selected sample of six to ten body feathers were taken from the body region of tagged individuals under licence from the UK Home Office. Data on trace metals in feathers collected from birds at the end of the winter in which they had been tracked were available from 46 birds. Concurrent data on C and N isotope data were available from 37 birds. Sex was determined by molecular analysis of DNA from feathers collected during logger recovery, using P2-P8 primer sequences and Z-002 and CAM-11 markers (Griffiths et al. 1998; Dawson 2008).

### Seasonal variation in activity patterns

Wet-dry sensors in the GLS loggers recorded whether tags were wet or dry every 3 s (see Mackley et al. 2011). Summary data on the number of wet samples in each 10 min period were then stored, allowing each 10 min period to be classified as wet, dry or mixed. These data were then used to explore seasonal patterns in the mean proportion of time spent dry in each month, to provide an indication of when flight performance may be constrained by the moult. Activity data were available for 68 individuals [post-breeding season (Sept–Dec): 33 females, 35 males, complete non-breeding season (Sept–May): 26 females, 29 males].

### The timing of feather moult

Data on feather moult were collected from carcasses during a long-term study of plastic contamination (van Franeker et al. 2011). Full details of the techniques used can be found in the Fulmar Litter EcoQO Manual Part 1: Collection and dissection procedures (van Franeker 2004). Primary and tail moult scores were recorded for each bird using criteria developed by the British Trust for Ornithology (BTO) (Ginn and Melville 1983). Moult scores were based upon external inspection of the feather, where an old feather was scored as 0 and a new, fully developed feather as 5. The maximum primary moult score for a fulmar was therefore 100, as fulmars have a total of 20 primary feathers. The maximum tail moult score was 70, as fulmars normally have 14 tail feathers. For the purposes of this study, April 1st was used as the reset date for the completion of moult, after which new plumage (total score 100 or score 70) must become old plumage (score 0) before the next moult cycle. Body feather moult was scored by internal assessment of the degree of active moult in body feathers in the sternal region of the ventral feather tract. Actively moulting feathers were identified by the broad and soft whitish (sometimes somewhat bluish) feather shafts, differing from the sturdy and pointed pins of full grown feathers (see Supplementary material Fig. 1). Moult was then scored as: 0 = no new feather shafts present in left or right sternal feather tract; 1 = 1–4 new soft whitish feather shafts in either or each of left or right sternal feather tract; and 2 = 5 or more new feather shafts present in either or each of left or right sternal feather tract section. Data were available from 725 adult fulmars which were defined as breeders based upon internal examination (see Supplementary material Table 1 for month by month sample sizes). These birds were either by-caught on Faroese or Icelandic long-lines, or recovered from local harvests in these areas, but were otherwise recorded as being in apparently healthy condition based upon body condition scores during dissection (van Franeker 2004). Mean moult scores for each feather type were calculated for each month of the year. These were then used to estimate the proportion of birds (with associated standard errors) that had completed moult (primary and tail, feathers) or were actively moulting body feathers, in each month.

**Fig. 1:**
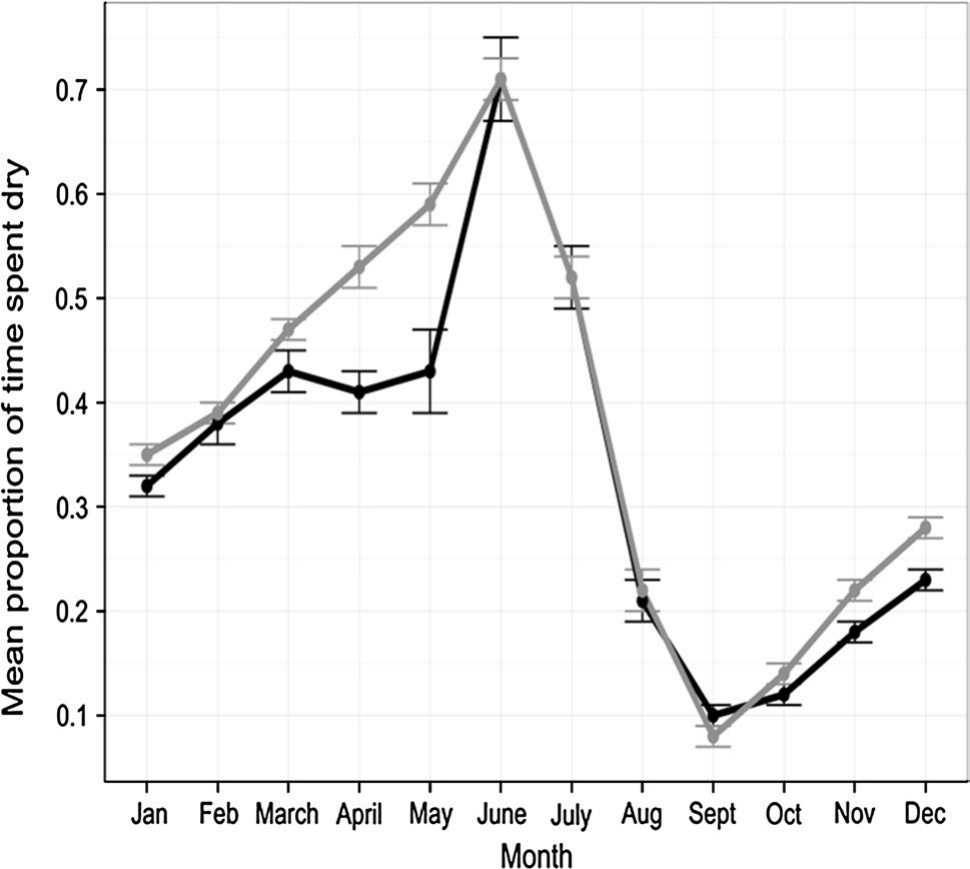
Variation in the mean proportion of time (±SE) that breeding fulmars spent dry in each month of the year. Data are based upon a sample of 32 females (*black*) and 37 males (*grey*) that had been instrumented with wet-dry activity loggers at the Eynhallow study colony

It is assumed for the purposes of this study that moult data from the adult birds caught off the Faroes and Iceland are representative of the moult pattern at North Sea colonies, including our main study site in Orkney.

### Chemical analyses of feathers

All feathers were first washed with Milli-Q water prior to oven drying at 65 °C for >12 h. For the trace metal analysis, aristar nitric acid (0.5 ml) was added to each feather sample and left overnight to digest. Aristar hydrogen peroxide (1 ml) was then added, before a 75 min microwave digest (CEM Microwave Technology Ltd.) in which a peak temperature of 95 °C was held for 30 min. Samples were then diluted to 10 ml with ultrapure deionized Milli-Q water, and 19 element total concentrations were measured from a sub-sample using an ICP-MS 7500 (Agilent Technologies), using the reaction cell with hydrogen gas. Bovine liver (National Institute of Standards and Technology) 1557b was used as the certified reference material (CRM). Standards made from multi-element solution 2 were run every 30 samples, from which standard curves could be calculated. In a few samples, some elements (V, Mn, As, Cd, Pb) were below the limits of detection (LoD). Following standard procedure (Anderson et al. 2010), these samples were given a value of half the LoD. All trace metal analyses were conducted at the University of Aberdeen.

For C and N isotope analyses, dried samples were homogenized in a ball mill (Mixer Mill type MM 200, Retsch of Haan, Germany) and sub-samples of 1.3 mg material loaded into 5 × 3.5 mm tin cups (Elemental Microanalysis Ltd.). Total N and C values and the ^15^N:^14^N and ^13^C:^12^C isotope ratios of milled dried material were determined using a Flash EA 1112 Series Elemental Analyser connected via a Conflo III to a Delta^Plus^ XP isotope ratio mass spectrometer (all Thermo Finnigan, Bremen, Germany). Isotope ratios were calculated using CO_2_ and N_2_ reference gasses injected with every sample. The isotopic values of these gasses were directly referenced against IAEA reference materials USGS40 and USGS41 (both l-glutamic acid); certified both for δ^13^C (‰_VPDB_) and δ^15^N (‰_air N2_). Long-term precision of a quality control standard (milled flour) was: δ^13^C 25.5 ± 0.29 ‰ and ^15^N 0.367 ± 0.0002 atom % (mean ± SD, *n* = 200). Samples of feathers from ten birds that were by-caught on Faroese long-lines were used to assess within-individual variation in chemical composition using four different types of feathers: primary (inners), secondary (opposite primaries), tail (central rectrices) and body (selection) feathers. Average sample weights were: primary (0.043 ± 0.009), secondary (0.040 ± 0.006), tail (0.051 ± 0007) and body (0.025 ± 0.003). Data were available for associated trace metals for between-feather comparisons, but not for C and N isotopic data. All stable isotope analyses were conducted at the James Hutton Institute.

Median concentrations of all elements above the limits of detection (LoD) were calculated for each feather type. Due to unequal variances between feather types, data were ranked (Langin et al. 2007) before performing repeated-measure ANOVAS. Feather type was the explanatory variable in the ANOVA, with each metal (loid) tested separately as the response variable. Individual bird ID was included as an error term to account for non-independence of individuals with repeated measures of feather types. Model fits were verified and pair-wise comparisons were made between each feather type using Tukey’s honest significant difference tests.

Between-feather variability in the chemical composition of body feathers was assessed using independent analysis of four or five feathers from ten individual birds that were sampled from the Eynhallow breeding population. For comparison with individuals in which measurements were based solely upon analyses of two body feathers, two body feathers were randomly sampled from each of these ten birds and the mean value calculated for each element (mg/kg). This process was then repeated for another two body feathers, and the two means were compared using the Pearson’s product moment correlation coefficient.

### Identification of non-breeding areas

For the sample of Eynhallow breeders that were successfully instrumented with GLS loggers, twice-daily light-based geolocations for each individual were produced using BASTrak software v.18. A light threshold of 10 and an elevation angle of −3.5 were used, prior to an iterative smoothing process that was applied twice to the data to reduce location errors (Phillips et al. 2004).

A mean location was then calculated for each of these individuals over the entire winter period between the equinoxes (October–February) and projected in ArcGIS v.9.3 using the North Pole Lambert Azimuthal Equal Area projection. These data were then used to assign each individual bird to one of the two broad-scale wintering regions (oceanic Atlantic waters and more nearshore waters over the continental shelf). If data from an individual bird were available from multiple years, only 1 year was randomly chosen for analysis to maintain independence.

For the 46 birds with both GLS tracking data and data on trace metals in body feathers, a MANOVA was carried out on all elements against location group to assess for differences in trace metal (loid)s between location groups.

For the 37 birds with a full suite of feather chemistry data, we then explored whether the trace metal and isotopic signatures of body feathers collected upon recapture could be used to identify the wintering region that each of these individuals had used. A linear discriminant analysis (lda) was carried out in the ‘MASS’ package in R (Venables and Ripley 2002) using trace element and stable isotope data from body feathers. Cross-validation techniques were employed to produce jackknifed predictions (Leat et al. 2013) which assigned birds to one of the two broad wintering regions with a given probability level. Initially, the discriminant analysis was run using data for each of the trace elements and both δ^13^C and δ^15^N stable isotope data. Based upon exploration of levels of variation between individual using the two wintering regions, these results were then compared with alternative models that used only C and N stable isotope ratios and finally, only N stable isotope ratios.

All statistical analyses were performed in R v.2.15.0 (R Development Core Team 2012), and statistical significance was taken to be *p* < 0.05.

## Results

### Seasonal variation in activity patterns

Activity data from GLS loggers demonstrated that September to December were the months in which individual birds spent the highest proportion of their time on the water (Fig. 1). Marked seasonal variation in individual activity patterns was seen in both males and females (Fig. 1), but there were slight differences between the sexes in April and May co-incident with the pre-laying exodus and early incubation period. Both sexes spent the highest proportion of time on the water in September (90.5 and 91.5 % wet for females and males, respectively) and the least amount of time wet in June (29.1 and 29 % dry for females and males, respectively).

### Variability in moult timings and chemical concentrations in different feather types

**Fig. 2:**
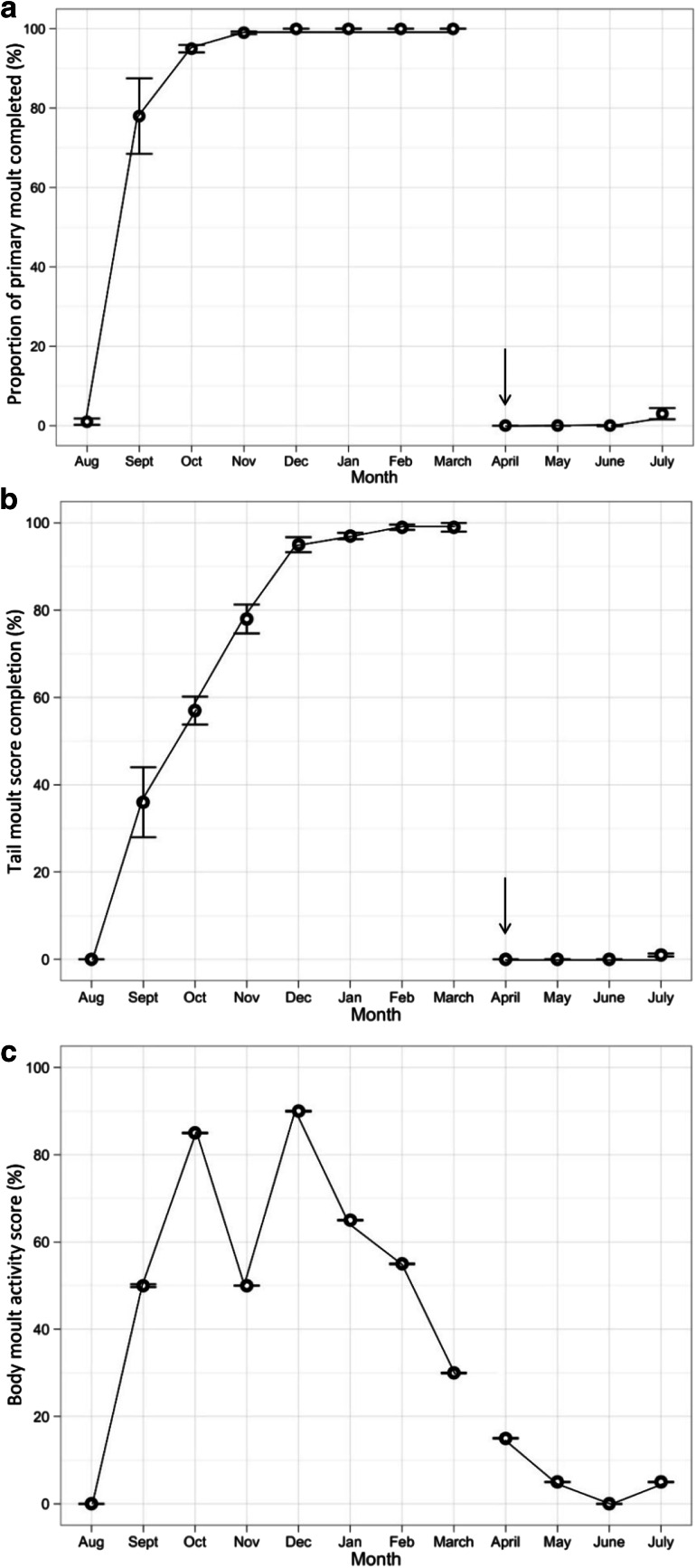
Variation in the proportion of adult fulmars sampled in each month that had** a** completed their primary moult (*n* = 704) and** b** completed their tail moult (*n* = 703). The* arrow* represents the reset date on April 1st. **c** Monthly variation in body moult activity, derived from the average monthly score for internal body moult expressed as a percentage of its maximum score of 2 (*n* = 725). Data are based upon analysis of apparently healthy harvested and by-caught birds from Faroese and Icelandic waters. Sample sizes are provided in Supplementary Table 1, and average moult scores for each feather type are provided in Supplementary Table 2

Analysis of moult scores revealed that primary moult in healthy adults occurred during September and October and was complete by November (Fig. 2). Little overlap with the breeding period in either wing or tail moult was recorded: the few initial phases of primary moult that were observed from June to August, were considered to represent an early start of moult amongst adults after breeding failure. Active tail moult in adults was seen over a longer period, from September to January, and was completed by February. Internal body moult was observed from September onwards, with the highest levels of active moult recorded in birds caught in October and December. However, these data indicate that active body moult occurs in adults throughout the non-breeding period (September to March) (Fig. 2).

Instrumental quality results for the chemical analyses demonstrated high percentage recovery for each element (Supplementary Table 3). In general, body feathers demonstrated smaller ranges in element concentrations than primary, secondary and tail feathers (Table 1). Fe and Zn, two essential elements, had the highest concentrations of all the elements studied and demonstrated large ranges (Table 1). All elements except As demonstrated a high degree of variability between feather types. Results of statistical comparisons of differences between the feather types revealed tail feathers had more significantly different pair-wise comparisons than any other feather type (9 of 13 elemental comparisons) and that primary and secondary feathers were similar in all but one element (V). Body feathers had similar metal (loid) concentrations to primary and secondary feathers in all but three elements (Supplementary Table 4). Tail feathers tended to have highest median concentrations of all elements compared to body, primary and secondary feathers, except for As, Se and Sr.

**Table 1:** Median concentrations of each trace element in mg/kg (range in brackets) in different feathers collected from a sample of 10 apparently healthy adult fulmars that had been by-caught in Faroese waters

Element	Feather type			
	Body median (mg/kg)	Primary median (mg/kg)	Secondary median (mg/kg)	Tail median (mg/kg)
V	0.21 (0.11–0.45)	0.433 (0.27–5.55)	0.28 (0.23–1.19)	1.56 (0.75–3.48)
Mn	0.92 (0.56–9.29)	1.74 (0.51–34.20)	1.92 (0.56–6.59)	4.79 (3.05–16.93)
Fe	131.72 (43.48–389.00)	97.71 (45.52–1440.04)	158.32 (32.79–2468.77)	777.30 (357.50–4215.83)
Cu	10.12 (8.55–11.62)	10.02 (7.94–77.95)	8.96 (6.35–13.97)	12.62 (9.85–27.51)
Zn	81.35 (59.84–102.26)	67.01 (51.93–184.87)	84.76 (68.35–127.88)	127.38 (44.36–326.22)
As	0.96 (0.48–1.92)	1.16 (0.33–17.61)	1.05 (0.24–3.01)	1.12 (0.46–6.74)
Se	3.87 (2.41–4.31)	5.23 (3.89–8.97)	5.64 (4.60–9.68)	3.44 (2.56–6.90)
Sr	15.03 (10.64–19.65)	30.82 (25.37–43.55)	29.15 (21.02–41.20)	28.21 (25.75–38.05)
Cd	0.22 (0.13–1.08)	0.16 (0.04–0.37)	0.11 (0.03–1.07)	0.47 (0.26–1.12)
Pb	0.53 (0.41–1.41)	1.35 (0.25–4.84)	0.66 (0.25–4.63)	4.36 (2.71–22.16)

Coefficients of variation were sometimes high when multiple body feathers were measured from the same bird (Table 2). However, variation between individuals was generally much greater. Thus, when the means of two randomly selected pairs of feathers from the same individual were compared, there was a positive, significant relationship between repeat measurements (Table 2).

**Table 2:** Mean concentrations of elements (mg/kg) and coefficient of variation (CV; %) for samples of body feathers from 10 adult breeding fulmars from Eynhallow

Bird	Element																				Mean 1 versus Mean 2 correlation
	V		Mn		Fe		Cu		Zn		As		Se		Sr		Cd		Pb		
	Mean	CV	Mean	CV	Mean	CV	Mean	CV	Mean	CV	Mean	CV	Mean	CV	Mean	CV	Mean	CV	Mean	CV	
1015	0.35	23	17.85	39	193.74	28	8.95	9	54.09	8	0.17	42	0.10	11	5.91	20	0.03	7	1.17	67	*r* = 0.99
1136	0.22	3	1.70	11	113.67	6	9.29	14	52.46	10	0.06	69	1.27	6	7.08	13	0.02	4	0.71	95	*r* = 0.99
1153	0.15	9	0.91	58	38.69	43	7.08	8	47.90	21	0.05	65	1.14	12	6.72	22	0.02	7	0.85	38	*r* = 0.98
1367	0.15	20	1.27	19	53.42	47	7.25	10	59.52	15	0.08	56	1.29	14	7.13	16	0.06	39	1.15	89	*r* = 0.94
1597	0.47	13	8.87	26	172.58	14	8.04	4	48.76	11	0.44	36	1.01	8	6.71	8	0.06	45	1.06	25	*r* = 0.99
1631	0.17	7	1.06	23	40.88	21	11.07	8	48.03	7	0.03	7	1.14	9	10.64	23	0.02	4	0.99	28	*r* = 0.99
1854	0.23	16	1.16	14	116.83	18	8.30	19	50.29	11	0.04	28	1.33	11	7.26	27	0.03	28	0.94	41	*r* = 0.99
1876	0.18	14	1.25	40	82.82	23	9.17	7	52.91	13	0.03	8	0.94	15	5.60	16	0.02	14	1.01	40	*r* = 0.98
1880	0.25	7	1.23	15	85.96	15	10.25	8	63.15	29	0.18	102	1.68	32	6.20	25	0.26	85	1.25	18	*r* = 0.81
1890	0.31	69	7.59	50	191.49	85	8.63	18	62.13	22	0.20	75	2.03	9	7.83	33	0.03	38	0.77	30	*r* = 0.99

The similarity between the chemical signatures of two independent pairs of samples from each individual is reflected by the correlation co-efficient in the final column. All correlations were *p* < 0.05

### Identification of non-breeding areas from intrinsic markers in feathers

**Fig. 3:**
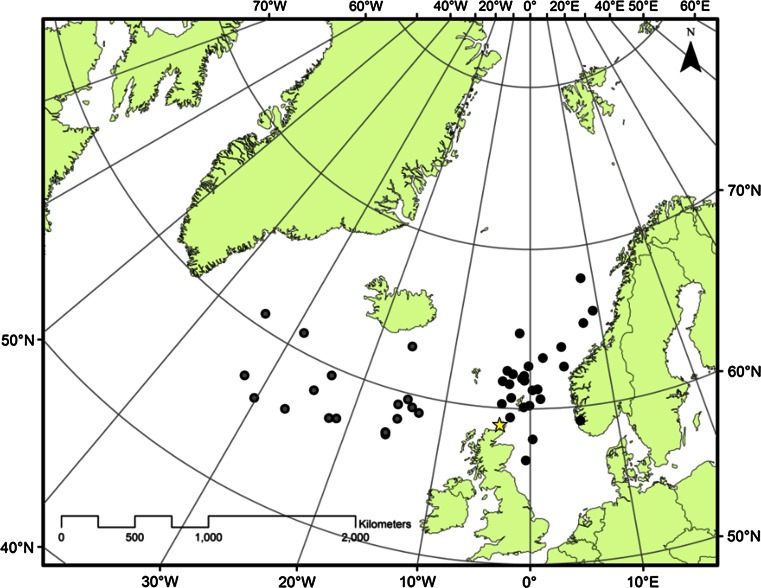
Mean winter location for the GLS tagged breeding fulmars for which data were also available on body feather chemical signatures. Mean winter locations are based upon daily locations obtained for each individual between mid-October to end of February. Based upon these mean locations, individuals were assigned to one of two broad wintering regions (Oceanic birds *grey*, Continental shelf birds *black*). The location of the Eynhallow breeding colony location is marked as a *star*

The sample of birds from which both GLS tracking and feather chemistry data were available included individuals that wintered over both Atlantic and continental shelf waters. The mean locations of each of these birds through the overall winter period (October–February) are shown in Fig. 3, together with the region that each bird was assigned to.

When considering each element separately, concentrations were similar between the group of birds wintering over the Atlantic and the group wintering over the continental shelf (Supplementary Table 5). Furthermore, there were no significant differences between groups when all elements were considered together (MANOVA, Pillai = 0.29, *F*_(9,36)_ = 1.61, Pr(>*F*) = 0.151).

There were also no significant differences in δ^13^C between location groups (ANOVA, *F*_(1,36)_ = 0.21, *p* = 0.649) but δ^15^N values were significantly different for birds wintering over the Atlantic and those wintering over the continental shelf (ANOVA, *F*_(1,36)_ = 53.11, *p* < 0.001) (Table 3).

**Table 3:** Median values of δ^13^C and δ^15^N isotope concentration in mg/kg (range given in brackets) for each winter location group: Oceanic Atlantic winterers (*n* = 14) and Continental Shelf winterers (*n* = 24)

Isotope	Oceanic median mg/kg (range)	Continental shelf median mg/kg (range)
δ^13^C	−17.18 (−17.87, −16.91)	−17.22 (−18.14, −16.13)
δ^15^N	14.27 (13.93, 15.46)	15.69 (14.81, 17.00)

**Fig. 4:**
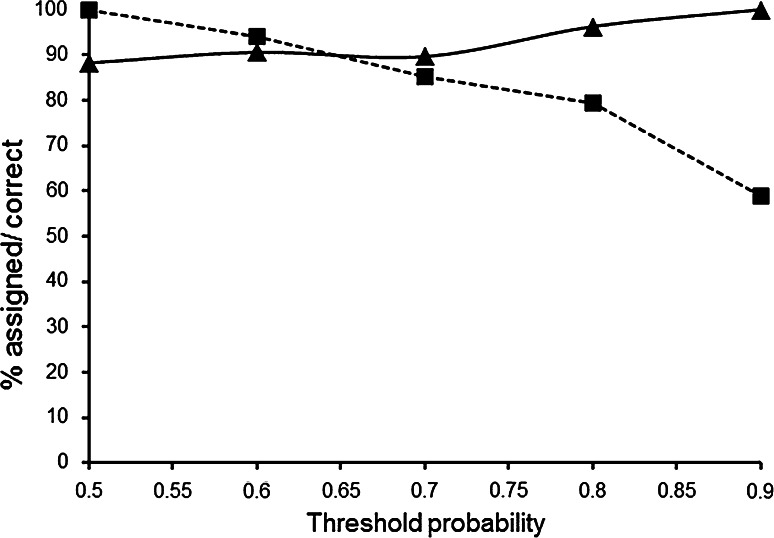
Variation in the percentage of birds that could be assigned to one of the two wintering regions from their feather isotope ratios (*filled square*) and the percentage of those assignments that were correct (*filled triangle*) in relation to the threshold probability used in the linear discriminant analysis

Using C and N isotope data, the discriminant analysis was able to correctly assign a high proportion (88 %) of birds to these different wintering regions (Table 4). Using the trace element data either alone or in combination with the isotope data resulted in lower predictive value (Supplementary Table 6). Raising the threshold for positive assignment reduced the number of individuals that could be assigned, but reduced the number of errors (Fig. 4). A cut off of 0.9 resulted in 100 % correctly assigned, but only 59 % of individuals could be assigned to a wintering region. Using a cut off of 0.8, 80 % of individuals were assigned to a wintering region, with a success rate of >95 %.

**Table 4:** Classification results from discriminant analysis carried out using two location groupings (oceanic and shelf winterers) with C and N isotopes

Allocated to group	True group	
	Oceanic	Shelf
Oceanic	11	2
Shelf	2	19
Total N	13	21
Proportion correct	0.85	0.90

## Discussion

This study has demonstrated that the chemical composition of body feathers can be used to make inferences about winter foraging ecology. Analysis of data from post-mortem investigations confirmed the time period over which body feathers grow in this species, thereby validating their use in diet and distribution studies over this period. Despite high inter-feather variability, mean values from pairs of body feathers provided a robust measure of individual variation in chemical loadings. These analyses, used in combination with tracking data from a sub-set of individuals, indicate that stable isotope markers in body feathers can provide insights into individual variability in the winter distribution patterns of breeding fulmars.

### Timing of moult

Activity data collected from breeding adults indicated that flight time was reduced during September and October (Fig. 1), supporting previous suggestions that flight feathers are moulted at this time of year (Dott 1973). These findings build upon recent work on Antarctic procellarids (Cherel et al. 2016) that highlighted the value of using activity data from GLS loggers to identify the timing of moult. The pelagic nature of this species has compromised efforts to confirm when and where adult fulmars from particular breeding colonies moult. Direct observations of feather moult have been restricted either to fulmars harvested or by-caught in the northern part of their range, or beached individuals that are more commonly recovered from the south of their range. Tracking data collected during this study highlight the extensive winter ranges of birds from our primary study colony (Fig. 3) and provide confidence in our assumption that data on moult patterns of healthy adults of breeding age that were harvested or by-caught in Icelandic and Faroese waters are representative of birds from UK colonies.

The direct observations of moult in this sample of fulmars indicate that body feathers moult over a longer time span than primary feathers (Fig. 2), supporting their use as a tool for assessing trophic patterns over the non-breeding period. A shorter period of primary moult post-breeding, in conjunction with a more protracted period of body feather moult, is seen in other similar-sized seabirds (Furness et al. 1986; Bridge 2006). Wing moult constrains flight ability (Hedenström and Sunada 1999; Bridge 2006) and minimizing this period may be required to increase foraging opportunities at this critical time of year. In contrast, a longer period of body moult should not impede flight capabilities. Our data revealed no overlap between the breeding period (April to August) and either wing or tail moult initiation, confirming results from studies of fulmars breeding in the Canadian High Arctic (Allard et al. 2008). Similar patterns are found in other terrestrial and marine birds (Weimerskirch 1991; Hemborg et al. 2001), though some exceptions in the extent of overlap between breeding and moult do exist (see Barbraud and Chastel 1998; Ramos et al. 2009).

Chemical concentrations in body feathers were similar to those found in primary feathers (Table 1), suggesting the feathers were grown under similar conditions with respect to time, place or dietary intake. This concurs with other studies which demonstrated similarities in certain isotope levels between primary and breast feathers in wandering albatrosses *Diomedea exulans* (Jaeger et al. 2009) and common guillemots *Uria aalge* (Becker et al. 2007). In contrast, there were some differences between tail feathers and the other feather types, with tail feathers having higher concentrations of most elements. In fulmars, tail moult occurred in the latter half of winter, but this pattern of tail moult occurring after wing moult is not ubiquitous amongst other petrel species (see Hedd and Montevecchi 2006). Observed differences in chemical loadings in tail feathers could result from biological factors, such as differences in moult pattern or area; or fundamental differences in the sequestering of elements in tail feathers. Alternatively, differences may result from methodological issues related to feather preparation and external contamination of tail feathers. Tail feathers have a more complex structure than body feathers, and it is possible that, despite the Milli-Q water washing stage, external dust may have remained embedded in the feather (Font et al. 2007). External contamination of certain elements, commonly Fe, Hg, Ni and Zn, has been suggested in other feather studies (Dauwe et al. 2003) and the presence of heavy metals in preen oil (Goede and De Bruin 1984; Jaspers et al. 2008) may have affected chemical loadings in tail feathers. Whatever the cause of these differences, these data suggest that more detailed comparison of feather chemistry should avoid using tail feathers.

### Within-individual variability in the chemical composition of body feathers

Due to their less complex structure, the chemical loadings of body feathers may be less likely to be influenced by external contamination. Whole body feathers can also be collected non-intrusively during routine handling of live birds, and they have been used as a tissue of choice in a wide variety of seabird studies (Bearhop et al. 2000; Cherel et al. 2006). However, in contrast to studies where specific regions of a clearly defined wing or tail feather are sampled (Barrett et al. 2007; Kouwenberg et al. 2013), analyses are typically based upon a small number of randomly sampled body feathers. Interpretation of between-individual differences in chemical signatures from these samples must therefore be placed in the context of within-individual variability. Existing work on within-individual variation in chemical loadings has generally compared different feather types (Thompson and Furness 1995; Deben et al. 2012), but two studies have previously focused on variation within body feathers. Jaeger et al. (2009) considered the advantages and disadvantages of using an averaged value for a pooled sample of body feathers compared to average values from feathers subjected to independent analysis, whilst Carravieri et al. (2014) compared individual variation in body feathers from seabirds with synchronous and asynchronous moult patterns. As seen by Carravieri et al. (2014) in other seabirds with a more protracted moult, our analyses also found a high level of within-individual variation in levels of some trace metals in fulmar body feathers. For example, Fe varied markedly between feathers, as shown in other seabird studies (Jerez et al. 2011). Nevertheless, there were strong positive correlations in the overall trace metal signatures of paired independent samples of body feathers from the same individual, supporting further use of this easily sampled tissue in biogeochemical studies of northern fulmars.

As reported in previous studies of large procellarids (Phillips et al. 2009) and Great skuas *Stercorarius skua* (Leat et al. 2013), our integration of feather chemistry and tracking data indicated that C and N isotopes in body feathers provide a good indication of each individual’s wintering region (Table 4). Generally, it has been C isotopes in feathers that have been most informative given the underlying geographical variation in C isotopes in the marine environment (Cherel and Hobson 2007). However, in our study, all the discriminatory power came from the variation in N isotopes and there was no difference in C isotopes between birds wintering in different regions. Consequently, observed geographical variation in these fulmars could result from differences in the foraging strategies of birds using different regions, with those individuals occurring over oceanic waters foraging at lower trophic levels (Table 3). Alternatively, δ^15^N values at the base of the food chain may differ in these different regions. Further work is now required to discriminate between these alternatives, as this has important implications for understanding the nature of the variation in non-breeding strategies observed in this species.

Using δ^15^N values either alone or in combination with δ^13^C, 88 % of birds were correctly assigned, whereas using additional information from the trace metal analysis decreased our ability to predict wintering regions. However, it remains important to explore the value of a broad suite of markers when applying this approach in different systems. For example, Gómez-Díaz and González-Solís (2007) found that trace metals gave greater discriminatory power than stable isotopes when assigning other *Procellariformes* to different Atlantic and Mediterranean colonies. In our study, restricting the number of birds assigned by introducing a threshold for the bootstrap probability resulted in lower errors (Fig. 4), and future studies using this approach should carefully balance the need to maximize the proportion of birds assigned with the level of confidence in those assignments.

### Implications for future work

Intrinsic biogeochemical markers and tracking technologies provide increasing potential for studies of migratory patterns and foraging ecology. The integration of these techniques can be especially valuable, providing direct estimates of distribution and indirect information on diet and contaminant exposure in those areas. Body feathers provide an easily sampled tissue that can underpin these studies, but data interpretation can be constrained in many seabirds where there is limited information on moult patterns (Bridge 2006). Our study highlights that body feathers from fulmars can act as robust proxies for identifying individual variation in location and dietary patterns during the non-breeding period. Carefully designed feather sampling regimes can therefore provide a non-lethal sampling technique to improve our knowledge of seabird foraging during the non-breeding period. Some of these opportunities will be realized through biogeochemical studies that are integrated into the growing number of seabird tracking studies. It would be especially valuable to extend the study to other North Atlantic and North Pacific fulmar colonies to explore the extent to which this approach can be used in other regions. Other opportunities could result from the availability of much larger samples of feather samples compared to most tracking studies, and the availability of both contemporary and historic samples (e.g., Thompson et al. 1995). One important consideration is that study individuals need only be captured once to obtain a feather sample, whereas most tracking studies require a re-capture to retrieve a data logger (Hobson and Norris 2008). Recent tracking studies of a variety of seabird species have revealed that there is often within colony variation in wintering strategies, but the extent to which these may influence other aspects of behaviour that could bias tag retrieval remains uncertain. Our confirmation that body feather isotopic signatures reflect different wintering strategies at this UK colony now provide an opportunity to evaluate this bias, and further explore the nature of these alternative strategies and their population consequences.

## Electronic supplementary material

Below is the link to the electronic supplementary material.

Supplementary material 1 (PDF 414 kb)
