# Supplementary material for: Validating the use of intrinsic markers in body feathers to identify inter-individual differences in non-breeding areas of northern fulmars
Source: Mar Biol. 2016 Feb 29;163:64. doi: 10.1007/s00227-016-2822-1 (PMC4771816; doi:10.1007/s00227-016-2822-1)
Supplement: Supplementary file 1 — Supplementary material 1 (PDF 414 kb) [file 227_2016_2822_MOESM1_ESM.pdf]

Quinn et al. Validating the use of intrinsic markers in body feathers to identify inter-individual differences in non-breeding areas of northern fulmars.

Supplementary Material

Figure S1. Photographs of dissected Northern Fulmars a) showing evidence of active moult of body feathers, where many soft whitish feather shafts of growing feathers can be seen in both left and right sternal region *(score 2 = 5 or more growing feathers in each of the sternal regions).* b) showing no evidence of moult of body feathers: no soft whitish shafts of growing feathers present among the sturdy pins of fully grown feathers. *(score 0).*

a)

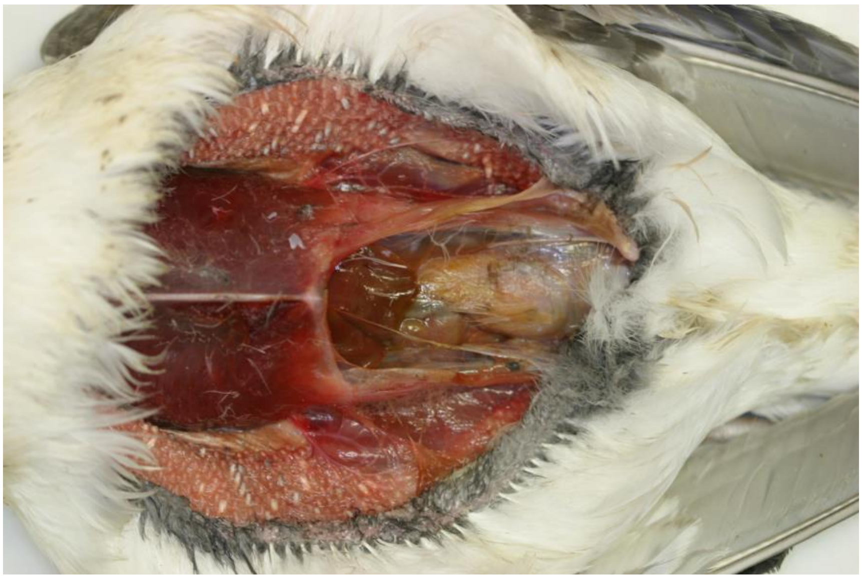

b)

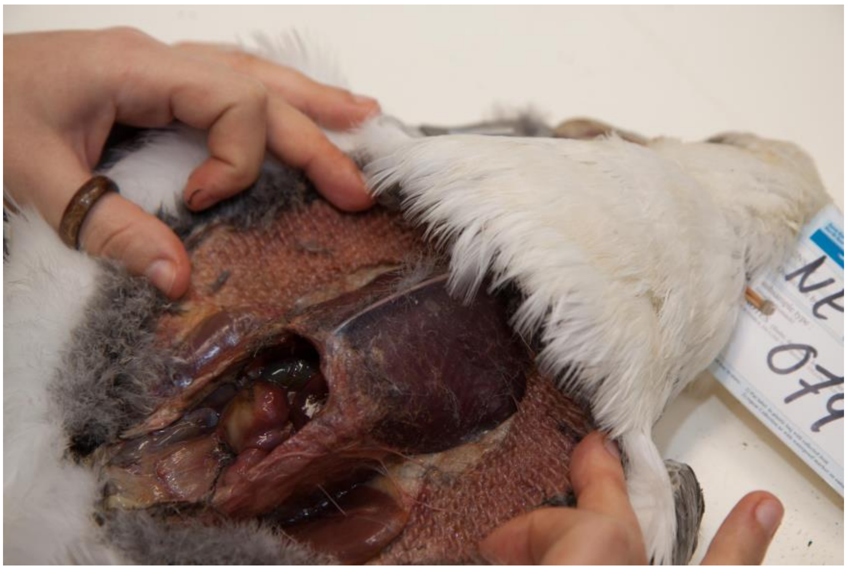

Table S1 Numbers of adult fulmars from the Faroe Islands and Iceland, sampled per month for moult data study in each feather type.

**Month**	**Primary** (no. of birds)	**Tail** (no. of birds)	**Body** (no. of birds)
January	103	103	102
February	78	78	77
March	45	45	45
April	107	106	104
May	104	104	130
June	17	17	17
July	100	100	100
August	11	11	11
September	10	10	10
October	61	61	61
November	30	30	30
December	38	38	38
**Total**	704	703	725

Table S2 Average moult scores for the sample of adult fulmars from the Faroe Islands and Iceland. Maximum moult scores varies for feather type: Primaries (100); Tail (70); Internal body (2). Sample sizes are in Table S1.

**Month**	**Primary**<br>Mean score (SE)	**Tail**<br>Mean score (SE)	**Body**<br>Mean score (SE)
January	99.9 (0.05)	67.9 (0.74)	1.3 (0.07)
February	100.0 (0)	69.4 (0.60)	1.1 (0.09)
March	100.0 (0.02)	69.0 (0.99)	0.6 (0.1)
April	0.0 (0)	0.0 (0)	0.3 (0.06)
May	0.0 (0)	0.0 (0.02)	0.1 (0.02)
June	0.1 (0.12)	0.0 (0)	0.0 (0)
July	3.1 (1.41)	0.4 (0.32)	0.1 (0.03)
August	1.2 (0.77)	0.0 (0)	0.0 (0)
September	77.5 (9.49)	25.1 (8.01)	1.0 (0.33)
October	95.1 (0.93)	39.6 (3.19)	1.7 (0.08)
November	98.6 (0.31)	54.7 (3.31)	1.0 (0.03)
December	100.0 (0)	66.3 (1.71)	1.8 (0.08)

Table S3 Limits of detection (LoD) for feathers samples (µg/g) and percentage recovery of the elements, using Bovine liver certified reference material (CRM) with the expected and obtained values.

**Element**	LoD (µg/g)	Expected CRM value (µg/kg)	Obtained CRM value (µg/kg)	% recovery for element
**As**	0.061	50	41	81
**Cd**	0.043	500	460	92
**Cu**	2.104	160000	143836	90
**Fe**	15.510	184000	175396	96
**Mn**	0.318	10500	9420	90
**Pb**	0.332	129	127	98
**Se**	0.174	730	856	117
**Sr**	0.794	136	221	163
**V**	0.088	123	119	96
**Zn**	4.097	127000	114089	90

Table S4. Results from repeated measure rank-based ANOVAs for each element/isotope ratio and feather type. Pairwise comparisons between feather type groups are also shown where B=body feather; P=primary feather; S=secondary feather and T=tail feather.

**Element/ Isotope ratio**	**Model**			**Pairwise comparisons between feather types that were significantly different**
	Feather type			
	F	*df*	*p*	
V	36.11	3	<0.001	All
Mn	15.71	3	<0.001	Tail vs B,P,S
Fe	9.53	3	<0.001	Tail vs B,P,S
Cu	9.95	3	<0.001	Secondary vs B,T
Zn	4.40	3	0.012	None
As	0.72	3	0.551	None
Se	40.15	3	<0.001	All except P&S,B&T
Sr	19.24	3	<0.001	Body vs P,S,T
Cd	8.32	3	<0.001	Tail vs P,S
Pb	18.58	3	<0.001	Tail vs B,P,S

Table S5. Median values of metal (loid) or isotope concentration in mg/kg (range given in brackets) for each location group. n=18 (Oceanic Atlantic), n=28 (Continental Shelf).

**Element/Isotope**	**Oceanic Atlantic**	**Continental Shelf**
	median mg/kg (range)	median mg/kg (range)
V	0.21 (0.09-0.80)	0.20 (0.09-0.50)
Mn	2.69 (0.32-17.21)	2.19 (0.32-29.4)
Fe	113.82(22.28-435.09)	102.57 (16.35-332.38)
Cu	9.03 (7.27-11.85)	9.57 (5.30-12.51)
Zn	60.04 (42.25-80.00)	58.91 (34.27-176.13)
As	0.16 (0.04-0.46)	0.11 (0.04-0.33)
Se	2.60 (0.03-12.59)	1.68 (0.84-2.74)
Sr	8.37 (5.94-12.70)	7.98 (3.96-22.10)
Cd	0.08 (0.03-0.42)	0.05 (0.01-0.48)
Pb	0.67 (0.38-8.31)	0.93 (0.27-2.37)

Table S6. Proportion of individuals correctly assigned to wintering regions defined from individual location data when using different combinations of chemical markers in the lda model.

	Oceanic Atlantic	Continental Shelf	Overall
$\delta^{15}N$ $\delta^{13}C$	0.9	0.85	0.88
$\delta^{15}N$	0.9	0.85	0.88
Trace metals (V, Mn, Fe, Cu, Zn, As, Se, Sr, Cd, Pb)	0.67	0.23	0.5
$\delta^{15}N$ + $\delta^{13}C$ + Trace metals	0.81	0.77	0.79
